# Nuclear Respiratory Factor 1 Acting as an Oncoprotein Drives Estrogen-Induced Breast Carcinogenesis

**DOI:** 10.3390/cells7120234

**Published:** 2018-11-27

**Authors:** Jayanta K. Das, Quentin Felty, Robert Poppiti, Robert M. Jackson, Deodutta Roy

**Affiliations:** 1 Department of Environmental Health Sciences, Florida International University, Miami, FL 33199, USA; jayantakdas.74@gmail.com (J.K.D.); feltyq@fiu.edu (Q.F.); 2 Research Service, Bruce W Carter VA Medical Center, 1201 NW 16th St, Miami, FL 33136, USA; Robert.Jackson4@va.gov; 3 Department of Pathology, Florida International University, Miami, FL 33199, USA; Robert.Poppiti@msmc.com

**Keywords:** estrogen, breast cancer stem cell, NRF1, oncoprotein, re-programming

## Abstract

We have previously shown nuclear respiratory factor 1 (NRF1)-mediated transcriptional programming of mitobiogenesis contributes to estrogen-induced breast cancer through modulating cell cycle progression. In this study, we report a new role of NRF1 that goes beyond that of programming mitobiogenesis. Specifically, we report a novel oncogenic function of NRF1 supporting its causative role in breast cancer development and progression. The gain of NRF1 and/or treatment with 17β-estradiol (E2) produced heterogeneous breast cancer stem cell (BCSC)-like subsets composed of more than 10 distinct cell sub-populations. Flow sorting combined with confocal imaging of markers for pluripotency, epithelial mesenchymal transition (EMT), and BCSCs phenotypically confirmed that the BCSC-like subset arise from cell re-programming. Thus, we determined the molecular actions of NRF1 on its target gene CXCR4 because of its known role in the acquisition of the BCSC-like subset through EMT. CXCR4 was activated by NRF1 in a redox-dependent manner during malignant transformation. An NRF1-induced BCSC-like subset was able to form xenograft tumors in vivo, while inhibiting transcription of CXCR4 prevented xenograft tumor growth. Consistent with our observation of NRF1-driven breast tumorigenesis in the experimental model, higher protein levels of NRF1 were also found in human breast cancer tissue specimens. This highly novel role of NRF1 in the stochastic acquisition of BCSC-like subsets and their progression to a malignant phenotype may open an entirely new research direction targeting NRF1 signaling in invasive breast cancer. Our discovery of targeting transcriptional activation of CXCR4 to inhibit NRF1-induced oncogenic transformation provides a mechanistic explanation for estrogen-dependent breast carcinogenesis and opens new avenues in strategic therapeutics to fight breast cancer.

## 1. Introduction

Nuclear respiratory factor 1 (NRF1) is widely recognized for regulating genes encoding mitochondrial biogenesis [1]. Recent evidence also indicates that the NRF1 protein interacts with a broad spectrum of transcription factors; its unique DNA binding recognition site is one of seven transcription factor binding sites most frequently found in the proximal promoters of ubiquitous genes [2,3,4]. The NRF1 motif is found on the promoters of genes regulating the cell cycle, chromatin structure, cell apoptosis, cell adhesion/invasion, DNA repair, DNA methylation and transcriptional repression signaling, and epithelial adherens junctions [2,3,4]. These findings suggest that NRF1 is a multifunctional protein with roles in diverse cellular functions. The actual role NRF1 plays in breast cancer remains the least studied of all transcription factors. We have previously reported the role of NRF1 in transcriptional programming of mitochondrial biogenesis in estrogen-induced growth of breast cancer cells [5,6]. Estrogen treatment increases mitochondrial mass, the DNA-binding activity of NRF1, a regulator of TFAM, and the level of TFAM, and TFAM shRNA inhibits colony formation in E2-treated breast cancer cells. These findings suggest that NRF1-mediated transcriptional programming of mitobiogenesis contributes to estrogen-induced cell cycle progression [5,7,8] Meta-analysis of 18 published breast cancer microarray data showed that NRF1 is elevated in high-grade breast tumors [7]. Our findings were validated by a recent study using TGCI normal and breast tumor specimens in which it was shown that NRF1 activity was significantly higher in human breast cancers compared to adjacent surrounding control breast tissue [8]. Furthermore, we have also shown that reactive oxygen species (ROS)-mediated activation of NRF1 is critical for the growth of estrogen-induced breast cancer cells and estrogen-induced malignant breast cell transformation [9,10]. Whether NRF1 contributes to estrogen carcinogenesis in breast cancer is not fully understood. Life time exposure to elevated levels of estrogen is a major risk factor for breast cancer [11]. Estrogen is a breast carcinogen; however, the molecular mechanisms responsible for estrogen-induced breast tumor initiation remain poorly understood. Although several signaling pathways may be targeted by estrogen in human mammary epithelial cells (HMECs) during the induction of a pre-malignant phenotype, our focus is on the NRF1 signal transduction pathway because DNA sequence motifs bound by NRF1 positively correlate with malignant breast cancer progression [12].

Despite tremendous progress in understanding breast cancer, gaps remain in our knowledge of the molecular basis underlying the aggressiveness of breast cancer. Breast tumor-initiating cells (BTICs) or breast cancer stem cells (BCSCs) are considered to be responsible for estrogen-induced initiation and aggressive progression of breast tumors [13,14]. We have recently shown that activation of the NRF1 pathway may participate in the development of breast tumors; however, its contribution to the acquisition of cancer stem cells remains unexplored in breast cancer [5,6]. This malignancy may occur via transformation of adult stem cells into cancer stem cells that give rise to the tumor. There are several key genes related to cell growth, cell transformation, cell adhesion/motility, and tumor suppression that are regulated by NRF1 [13]. Some of these genes, including CXCR4, are upregulated by estrogen treatment. The contribution of CXCR4 to reprogramming breast cancer cells to cancer stem cells is of particular interest to our research on NRF1. CXCR4 has an established role in the acquisition of BCSCs through epithelial mesenchymal transition (EMT) [14,15]. Thus, it is biologically plausible that high NRF1 activity in breast tissue increases susceptibility to estrogen-induced breast carcinogenesis via upregulation of the CXCR4 gene. This NRF1-regulated gene alone or in concert with others may contribute to the estrogen-induced malignant phenotype. The purpose of this study was to investigate whether NRF1-modulated CXCR4 expression drives estrogen-induced malignant transformation of breast epithelial cells to BCSCs and whether this NRF1 activity plays a major role in breast cancer development and progression. Our findings demonstrate a new oncogenic role of NRF1 that goes beyond that of programming mitobiogenesis. Overexpression of NRF1 combined with exposure to a carcinogenic dose of 17β-estradiol (E2) through regulating CXCR4-generated BTICs that formed tumors in vivo. Further clinical validation of this finding may lead to new avenues for NRF1 targeted therapeutic strategies to fight breast cancer.

## 2. Materials and Methods

### 2.1. Cell Lines

MCF-10A, MCF-7, and MDA-MB231 cell lines were obtained from American Type Culture Collection (ATCC, Manassas, VA, USA). MCF-10A and MD-MB 231 cells were transfected with vector or cMV-NRF1-GFP construct (RG220113, OriGene Technologies) or Myc-tagged DN NRF1 (a gift from Dr. Jian Fu). 

### 2.2. Antibodies and Immunoblotting Analysis

Analysis of protein expression was performed by immunoblotting as described previously [7,8]. Western blots and were probed with the following antibodies: NRF1 (Rockland Immunochemicals Inc., Pottstown, PA, USA), Nanog (Cell Signaling Technology, Inc., Danvers, MA, USA), Vimentin (cat# AF2105, R&D System, Minneapolis, MN, USA), E-Cadherin (cat# ab15148, Abcam, Cambridge, MA, USA), N-Cadherin (cat# sc-8424, D-4, Santa Cruz Biotechnology, Inc., Dallas, TX, USA), CXCR4 (cat# sc-9046, H-118, Santa Cruz Biotechnology, Inc., Dallas, TX, USA), ALDH1 (cat # sc-22589, L-15, Santa Cruz Biotechnology, Inc., Dallas, TX, USA), and β-actin (13E5, rabbit mAb #4970, Cell Signaling Technology, Inc.). Electrochemiluminescence (ECL) intensity of detected target proteins was imaged and quantified with a Bio-Rad Versa Doc instrument. All immunoblots were completed a minimum of three times for each experiment.

### 2.3. Fluorescence-Activated Cell Sorting (FACS) and Immunofluorescence Detection

For visualization of GFP in the NRF1-GFP expressing cells, we set the excitation wavelength at 395 nm and emission wavelength at 475 nm and used a green filter channel, whereas for visualization of FITC we chose a yellow bandpass filter on our flow cytometer that was centered on a 495 nm excitation wavelength and a 591 nm emission wavelength in order to capture photons from the FTIC fluorescence in this region of the spectrum to minimize/avoid the potential for GFP spectral overlap. Vector control and DN NRF1 cells that have no GFP were only positive for the FITC channel of CD24, indicating that we did not obtain 591 nm emission from 395 nm excitation. CD24, CD44, and CD49f expression was analyzed in cells derived from mammospheres following incubation in trypsin-EDTA or dissociation with a Pasteur pipette and passage through a 40 μm sieve. Cells were pelleted by centrifugation, resuspended in 15 μL of monoclonal mouse anti-human CD24-fluorescein isothiocyanate (FITC) antibody (cat # 130-099-118, clone: 32D12, Miltenyi Biotec, Auburn, CA, USA), monoclonal mouse anti-human CD44–phytoerythrin (PE) (cat# 130-095-180, clone: DB105, Miltenyi Biotec), CD49f antibody (cat# 130-107-831, REA518, Miltenyi Biotec), ALDH1 (cat # sc-22589, L-15, Santa Cruz Biotechnology, Inc.), with mouse anti-goat IgG-PE, (cat# sc-3752, Santa Cruz Biotechnology) and incubated for 45 min at 4 °C. Three independent experiments were performed. Co-expression of BCSC markers CD24+/CD44+/CD49f+/EpCAM+/ALDH1+/CD133+/CXCR4+ were analyzed by the Guava easyCyte TM flow cytometer (Milipore) with the CytoSoft software program according to the manufacturer’s instructions. For CD44+/CD24+/CD133+/ALDH1+ cells, the cells were resuspended with a sorting buffer and incubated with anti-CD24-FITC, anti-CD44-PE, anti-CD133-APC, and anti-ALDH1-QDot605 antibodies.

The cells were fixed with methanol, and immunofluorescence staining was performed according to standard procedures. In brief, cells were blocked with 3% normal goat serum and then incubated with antibodies: anti-NRF1, anti-CD44-FITC, anti-CD49f-FITC, anti-CXCR4, or anti-CD133 overnight at 4 °C. After washing, cells were incubated with anti-mouse-IgG Alexa Fluor^®^ 633 (Life Technologies Corporation, Carlsbad, CA, USA) for anti-CD133 and anti-rabbit-IgG Alexa Fluor^®^ 546 for NRF1 and CXCR4. Cells were mounted with Fluoromount-G™ reagent and analyzed using a Nikon C1 laser scanning confocal microscope. The MCF10A wild type and NRF1 cells were seeded on the wells of eight chambered slides with or without treatment of E2 (100 pg/mL) for 24 h.

### 2.4. MTT, SRB, and BrdU Assays

MCF-10A, MCF-7, and MD-MB 231 cells were seeded in 96-well plates at a density of 1 × 10^4^ cells/mL and treated with or without E2 (100 pg/mL) for 24 h. Methylthiazoletetrazolium (MTT) solution was added to each well and incubated for 30 min at 37 °C. Thereafter, the MTT solution was removed followed by the addition of DMSO to dissolve the formazan crystals. Absorbance was measured in a Tecan Genios plate reader at 560 nm (reference λ at 700 nm). For the SRB assay, cells were fixed with 50% trichloroacetic acid at 4 °C (50 μL/well) for 1 h. The plate was washed with tap water five times, dried, and stained with SRB dye (0.057% in 1% acetic acid) for 30 min and subsequently washed with 1% acetic acid to remove the unbound dye. The plate was air-dried, and the bound protein stain was solubilized with 100 μL of a 10 mM Tris base. The absorbance was measured at 540 nm using a Tecan Genios microplate reader. Bromodeoxyuridine (BrdU) incorporation was determined as a biological indicator of DNA synthesis in cells treated with E2 (100 pg/mL). Cells were grown (2500 cells per well) in 96-well plates and labeled with BrdU for 24 h. Afterwards, a colorimetric BrdU cell proliferation assay was performed according to the manufacturer’s instructions, as described previously [8].

### 2.5. FITC Annexin V Apoptosis Detection

Cell apoptosis was assessed by using FITC Annexin V Apoptosis Detection Kit I (BD Pharmingen, San Jose, CA, USA) according to the manufacturer’s protocol. Briefly, cells were washed with cold PBS and then resuspended in 1X binding buffer, and 100 µL of solution containing cells were stained with 5 µL of Annexin V-FITC and 5 µL of propidium iodide (PI). Cells were analyzed by the Guava easyCyte using the CytoSoft software program according to the manufacturer’s instructions.

### 2.6. Cell Invasion Assay

Cells from mammospheres (5 × 10^3^ cells/mL) were seeded in serum-free media in the upper chamber of a transwell insert; 10% FBS medium was added to the lower chamber as a chemoattractant. After 24 h incubation, invaded cells were stained with 0.2% crystal violet and images were acquired by microscope. Invasive cells were counted and scored from the lower chamber in triplicate.

### 2.7. Colony Formation Assay

Cells from mammospheres were seeded on top of soft agar (0.3%) with a bottom layer of 0.7% agar in DMEM/F12 in six-well plates and maintained in complete medium for 14 days. Cells were treated with DMSO as a vehicle or E2 (100 pg/mL). After 14 days, the cells were washed with PBS, fixed in methanol for 15 min, and stained with 0.005% crystal violet crystal violet for 15 min. The plates were then photographed, and the colonies were counted. At least three independent experiments were carried out for each assay.

### 2.8. Tumorigenic Spheroid Assays

For tumorigenic spheroid formation, approximately 100 cells per well were seeded in an ultra-low attachment 96-well plate (Corning Inc., Lowell, MA, USA). The tumorigenic spheroids were photographed with the HoloMonitoring and confocal microscopy as live images. Cells were suspended in serum-free DMEM/F12 (1:1) culture medium supplemented with B27. Approximately 100–150 cells per well were seeded in an ultra-low attachment 96-well plate (Corning Inc., Lowell, MA, USA). The effect of carcinogenic regimen of 17β-estradiol (E2) was evaluated by E2 treatment (100 pg/mL) on the day of seeding cells. Spheroids were grown for 27 days in liquid culture. A total of 15 spheroids with a minimum diameter of 50 mm were counted in each experimental group. Data were analyzed by ANOVA; Tukey’s HSD test was used for multiple comparisons. Cells obtained from spheroids were analyzed by immunofluorescence, FACS, or immunoblotting, as described previously [7,8].

### 2.9. Cell Differentiation

For differentiation, both an NRF1 MCF10A clone and the vector MCF10A were maintained in a chondrocyte differentiation medium (Sigma, St. Louis, MO, USA) for 28 days. The NRF1 MCF10A clone and vector MCF10A were cultured in either SMGS plus DMEM-F12 or neurobasal media plus nerve growth factor-7S (Sigma), Glutamax, and N2 supplement for a total of 28 days. We determined cell differentiation of NRF1 MCF10A clone at 28 days with staining of Alician blue, α-smooth muscle actin (smooth muscle marker), and β-tubulin III (neuron marker). Nuclei were counterstained with DRAQ5^®^ (Cell Signaling).

### 2.10. Cell Migration Assay

Cells were cultured in 6-well plates and a sterile plastic 1 mL micropipette tip was used to scratch in the middle area of the well as a line. Then cells were incubated in growth medium for 48 h. The scoring wounds were photographed with the HoloMonitoring and confocal microscopy as live images.

### 2.11. Detection of Senescent Cells

The Senescence β-Galactosidase Staining Kit (Cell Signaling Technology, Inc.) was used which provided the reagents needed to detect β-galactosidase activity, a known characteristic of senescent cells of spheroids. Single cells were obtained from the spheroids using 0.5% trypsin, and these cells were seeded on the wells of eight chambered slides for detection of blue colored beta-galactosidase activity as senescent cells. The cells were photographed with a compound optical microscopy.

### 2.12. Determination of Reactive Oxygen Species (ROS)

Single cells were obtained from the spheroids using 0.5% trypsin, and these cells were seeded on the wells of eight chambered slides for the detection of ROS by oxidation of 2′,7′-dichlorofluorescein-diacetate (DCFH-DA) (Molecular Probes, Eugene, OR, USA as described previously [7,8]. The oxidative products were visualized by a confocal microscopy.

### 2.13. Drug-Resistant Characteristic of Mammospheres

The MCF-10A, MCF-7, and MDA-MB-231 cells were suspended in serum-free DMEM/F12 (1:1) culture medium supplemented with B27. One hundred cells per well were seeded in an ultra-low attachment 96-well plate (Corning Inc., Lowell, MA, USA). The breast cancer chemo therapeutic drug registrant characteristic of mammospheres were evaluated by treating fulvestrant (5 µM) (I4409, Sigma-Aldrich, St. Louis, MO, USA tamoxifen (10 µM) (Sigma-Aldrich), and paclitaxel, (50 and 100 µM) (Sigma-Aldrich) with or without E2 (100 pg/mL) on the day of seeding cells. The mammospheres were photomicrographed and analyzed at Days 7 and 10.

### 2.14. In Vivo Tumorigenesis in Immunodeficient Mice

We prepared the cell suspensions for transplantation as follows: Approximately 80% confluence, CD24−44+133+ALDH+ MCF-10A stem cells were washed twice with phosphate buffered saline (PBS) and treated with 0.25% trypsin-EDTA solution (Life Technologies) for detachment from cell culture dishes. The CD24−44+133+ALDH+ MCF-10A stem cells of different groups namely vector plus E2 (100 pg/mL), NRF1, NRF1 plus E2 (100 pg/mL), NRF1 plus E2 (100 pg/mL) plus control CXCR4, and NRF1 plus E2 (100 pg/mL) plus siRNA CXCR4 were counted (1 × 10^3^) and prepared in 100 μL of a 1:1 (*v*/*v*) mixture of cell culture medium and Matrigel for transplantation.

Immunodeficient female NOD/SCID mice (Jackson Laboratory) were maintained in the animal research facility of Miami VA Healthcare System and were used for in vivo tumorigenicity studies. Prepared cell suspensions were injected using 1 mL syringes with a 25 G needle (Terumo, Shibuya, Tokyo, Japan) into mammary fat pads of 8-week-old mice (*n* = 5). The mice were palpated weekly for 6 weeks to observe nodule formation at the injection site. The successive engraftment was determined according to progressive nodule growth at the injection site. Mice were humane euthanized and sacrificed at 42 days (6 weeks). The tumors were weighted with a digital balance. The protocol of the present study was reviewed beforehand and approved by the Institutional Animal Care and Use Committee (IACUC) of the Miami VA Healthcare System. All animal experiments were performed according to the Ethical Guidelines for Animal Experimentation from the VA IACUC. All animals were sacrificed under humane euthanasia with carbon dioxide inhalation and all efforts were made to minimize suffering. The tumors were isolated and fixed with 10% neutral buffered formalin. The paraffin-embedded sections were investigated by H&E staining for histological analysis.

### 2.15. Chromatin Immunoprecipitation (ChIP) qPCR Assay to Analyze NRF-1 Binding to the Promoters of CXCR4 Genes

Chromatin immunoprecipitation assays (ChIP) were carried out with Epitech Chip qPCR primer assay (Qiagen, Germantown, MD, USA). The MCF10A cells of vector, NRF1+, NRF1−(dominant negative for NRF1) were treated with or without E2 (100 pg/mL), for 24 h and analyzed by ChIP assay using the anti-NRF-1 antibody. The CXCR4 promoter region (−109 bp to −98 bp) in the NRF1 precipitated chromatin was amplified by real-time PCR using Epitech Chip qPCR primer assay for human CXCR4 NM_001008540.1 (-)03Kb Cat # GPH1021572(-)03A and Epitech chip one day kit according to the manufacturer’s (Qiagen Science, Inc.) instructions. Chromatin immunoprecipitation qPCR results were calculated using the ΔΔ Ct method.

### 2.16. Luciferase Reporter Assay for Active CXCR4 Gene Promoter

Cells were seeded in a 6-well dish and transfected with desired plasmids using Lipofectamine 2000 reagent (Invitrogen, Carlsbad, CA, USA ). Cells were treated with DMSO or E2 (100 pg/mL). The assays were performed with CXCR4 luciferase reporter (pLightSwitch Prom, Switch Gear Genomics, Inc., Carlsbad, CA, USA) using the manufacturer’s luciferase assay reagent. The cells were harvested after 24 h. Each data point obtained is the mean of three independent experiments.

### 2.17. Real-Time qRT-PCR Analysis for Detection of CXCR4 mRNA Levels

Total RNAs were isolated with TRIzol reagent from MCF10A cells of each group namely vector, NRF1+, NRF1− (dominant negative for NRF1) exposed to DMSO or E2 (100 pg/mL). RNA sample was reverse-transcribed into cDNA using the RT2 First Strand Kit from SuperArray Bioscience Corporation, Qiagen (Frederick, MD, USA) according to the manufacturer’s protocol. The polymerase chain reaction (PCR) reactions using cDNA were performed in an Applied Biosystems 7300 Real-Time PCR System using RT2 SYBR Green/ROX qPCR Master Mix and the manufacturer’s thermal cycler protocol with 2 primers (Catalog No. PPH00621A-200, Gene Symbol: CXCR4, bp: 1912, Ref Seq Accession No: NM_001008540) for CXCR4 and with 2 primers (Catalog No. 330001 PPH00073E, Gene Symbol: ACTB, bp: 191, Ref Seq Accession No: NM_001101.3) for β-actin (SuperArray Bioscience Corporation, Qiagen). CXCR4 was quantitated in triplicate for each sample and was determined by a “delta Ct and delta–delta Ct” calculation with reference to the housekeeping gene β-actin control. Results represent the means of three independent experiments performed in triplicate.

### 2.18. Immunofluorescence Study for CXCR4, 8-oxo-dG, and Real-Time qRT–PCR Analysis for CXCR4mRNA with Treatment of ROS Scavengers

Cells were pretreated for 4 h with ROS scavengers 20 μm ebselen (Eb) or 1 mM N-acetylcysteine (NAC) (Sigma), followed by treatment with E2. Antibodies for CXCR4 and anti-8-hydroxydeoxyguanosine (8-oxo-dG) (mouse mAb, Trevigen, Inc., Gaithersburg, MD, USA) was used for immunofluorescence study. The total RNA sample was reverse-transcribed into cDNA using the RT2 First Strand Kit from SuperArray Bioscience Corporation, Qiagen, followed by PCR reactions using cDNA, RT2 SYBR Green/ROX qPCR Master Mix with primers for CXCR4 (bp: 1912) and with primers for ACTB (bp: 191). CXCR4 was quantitated in triplicate for each sample and was determined by a “delta Ct and delta-delta Ct” calculation with reference to the housekeeping gene β-actin control. Results represent the means of three independent experiments performed in triplicate.

### 2.19. Statistical Analyses

All statistics were performed using VassarStats statistical software (Richard Lowry, Poughkeepsie, NY, USA). One-way analysis of variance (ANOVA) was performed to detect any differences between groups. If the result of the ANOVA was significant, pair wise comparisons between the groups were made by a post-hoc test (Tukey’s HSD procedure).

## 3. Results

### 3.1. Normal Breast Epithelial Cells Acquire Breast Tumor Initiating Properties by a Gain in NRF1 Activity in Conjunction with Exposure to E2

**Figure 1:**
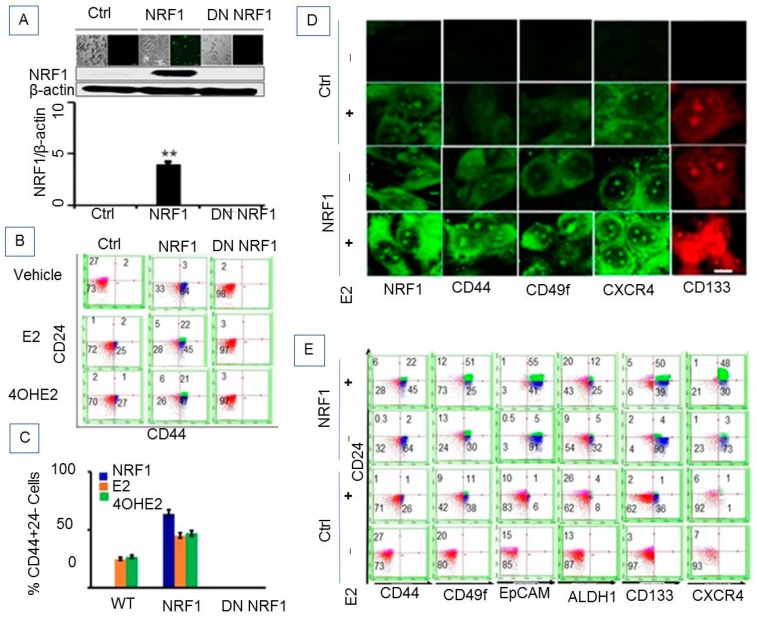
Heterogeneric subpopulations of estrogen-induced tumor initiating breast cancer-like cells. (**A**) Confirmation of NRF1 overexpression in MCF-10A cells after 14 days of transfection of cMV-NRF1-GFP construct (RG220113, OriGene Technologies, Rockville, MD, USA) detected by GFP expression by Nikon confocal microscopy (upper panel) and confirmed by Western blotting probing with anti-NRF1 antibodies (middle panel) and their subsequent densitometry analysis of NRF1 in relation to β-actin (bottom panel). Cell seeding was captured by phase contrast imaging (upper panel). ** *p* < 0.01 vs. control. (**B**) The representative flow cytometry data analysis showing effects of E2 and 4-OHE2 treatment to MCF10A cells on the expression of breast cancer stem cell markers CD24 and CD44. (**C**) The line graph represents the relative CD24 and CD44 co-expression in E2- and 4-OHE2-treated cells compared to vehicle control cells from three independent experiments. (**D**) Immunofluorescent detection of tumor initiating stem cell markers (NRF1, CD44, CD49f, CXCR4, and CD133) in E2-treated MCF10 A cells (Scale = 70 µm, 650X). (**E**) The representative flow cytometry showing further sorting of the CD24−/(low)/CD44+ cell population for expression of other BCSC markers—ALDH1A1, CXCR4, NRF1, and CD133.

We have shown that estrogen-induced malignant breast tumor formation is NRF1-dependent [9]. It was therefore logical to test whether the combination of NRF1 overexpression along with a carcinogenic E2 treatment regimen could generate BTICs. We used a spontaneously immortalized human breast epithelial MCF-10A cell line, derived from breast tissue of a woman with fibrocystic disease that is phenotypically normal [16]. Estrogen receptor (ER) α protein is not detectable in these cells. We chose this well characterized MCF-10 cell line, because we have already shown that E2 or its hydroxy metabolite, 4-OH-E2, transforms MCF-10A cells [9]. Stable NRF1 overexpressing MCF10A cells, after treatment with a carcinogenic regimen of E2, were grown as mammospheres in B27 medium then flow cell sorted for the breast cancer stem cell signature CD24−/CD44+. MCF10A cells showed stable ectopic expression of NRF1 and dominant negative NRF1 confirmed by immunofluorescence detection and Western blot (Figure 1A).

#### 3.1.1. E2 Treatment Enhanced Breast Tumor-Initiating (Breast Cancer Stem) Cell Features

Flow cytometry analysis of normal breast epithelial MCF10A cells (wild-type or CMV vector) indicated two distinct cell subpopulations based on differences in the expression of CD24 and CD44 markers. As shown in Figure 1B,C and Table 1. 

#### 3.1.2. Ectopic NRF1 Expression Enriched the CD44 (High)/CD24 (Low) Progenitor Subtype in Mammary Epithelial Cells and Increased E2-Induced Progenitor Subpopulations

E2 treatment increases NRF1 expression [18]. Recently we have shown that NRF1 expression was increased in MCF10A cells upon E2 exposure and that estrogen-induced breast cancer cell growth was reduced by the inhibition of NRF1 expression [9]. Based on our previous studies, we determined whether NRF1 contributes to the generation of cancer stem cells during E2-induced cell transformation of breast epithelial cells. Stable NRF1 overexpression resulted in a low percentage of CD24+/CD44+ (2.36%) cells and a higher percentage expressing the CD24−/CD44+ (64%) subtype (Figure 1B,C and Table 1). MCF10A cells were sorted for a population of CD24− or low/CD44+ tumor-initiating cells because this subtype represents tumor-initiating properties in breast cancer [19,20]. These CD24−/CD44+ cells (shown in bold in Table 1) were then treated with a carcinogenic regimen of E2 or 4-OH-E2 and propagated as mammospheres for the following experimental groups: wild-type (WT), vector, NRF1 overexpression, and dominant negative NRF1. Ectopic NRF1 expression changed the percentage of CD24+ and CD24−/CD44− subtypes in vector control MCF10A cells from 27.46 to 0.26% and 72.54 to 33%, respectively. These results suggest that both of these subpopulations were re-programmed to the CD24+/CD44+ and CD24−/CD44+ phenotypes.

Ectopic NRF1 expression in MCF10A cells when exposed to carcinogenic E2 treatments showed an increase in acquiring the CD24+/CD44+ subtype (2.36% in NRF1 treatment alone was enriched to 21.54% in NRF1+E2) (Figure 1B,C and Table 1). We also observed a decrease in CD24−/CD44+ subtype from 64% in NRF1 alone to 44.54% in NRF1+E2. Dominant negative (DN) inhibition of NRF1 completely suppressed the induction of CD44+ cells (Figure 1B). In summary, a gain in NRF1 expression and/or treatment with E2 led to reprogramming of normal breast epithelial cells to acquire the breast cancer stem cell signature CD24−/CD44+.

#### 3.1.3. Phenotypic Characteristics of Estrogen-Induced Tumor Initiating Breast Cancer Cells

**Figure 2:**
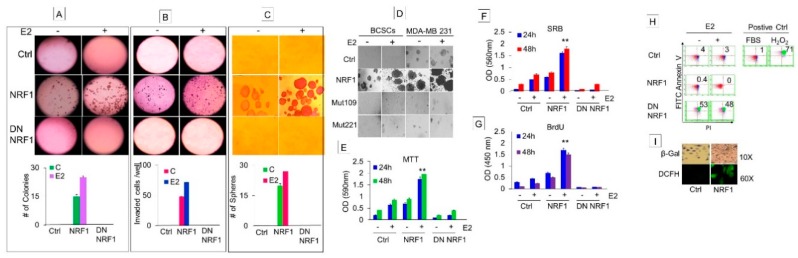
Phenotypic characteristics and growth properties of estrogen-induced tumor initiating breast cancer cells. Phenotypic properties of transformed breast epithelial cells of MCF10A [MCF-10A (T); breast tumor initiating cells with CD24 and CD44 signature (BTICs); breast cancer stem cells (BCSCs)] overexpressing vector (control), NRF1, or DN NRF1 treated with DMSO and E2 were assessed for anchorage-independent growth (**A**), migration and invasion (**B**), and mammospheres (**C**). Bottom panels of A, B, and C show quantitative changes in colonies, invaded cells and mammospheres in vector, NRF1 and DN NRF1 treated with DMSO (control) and E2 at 14 days, respectively. (**D**) Inhibition of mammosphere formation of BCSCs by NRF1 mutants 109 and 221 at 14 days. Growth of BCSCs overexpressing vector (control), NRF1, or DN NRF1 treated with DMSO and E2 measured by assessing metabolic capacity by the MTT assay (**E**); cellular protein content by sulforhodamine B (SRB) (**F**); DNA synthesis by BrdU incorporation assay (**G**). FACS analysis after FITC Annexin V (*X*-axis) and propidium iodide (PI: *Y*-axis) staining for apoptosis (**H**) and senescence by SA-β-gal activity histochemically (**I**) of BCSCs overexpressing vector (control), NRF1, or DN NRF1 treated with DMSO and E2. Error bars represent the mean of three independent experiments ± SD. ** *p* < 0.01 vs. control.

Self-renewal capacity of BTICs expressing the breast cancer stem signature was determined by measuring colony formation that also included measurements of cell motility and invasion. BTICs generated by the gain of NRF1 alone showed anchorage-independent growth, migration, and invasion, and formed mammospheres when compared to the control (Figure 2). When these BTICs were further exposed to E2, we observed an increase across all of the same phenotypic properties. These phenotypes were not detectable in CD24−/CD44− MCF10A control cells. Suppression of NRF1 by DN NRF1 inhibited NRF1 and/or E2-induced anchorage-independent growth, invasion (Figure 2A,B), and tumorosphere formation of BTICs (Figure 2C). We also observed that phosphorylation-deficient NRF1 mutants (mutation at amino acid residues 109 and 203 of the NRF1 protein sequence) suppressed the susceptibility of the BTICs to develop tumor spheroids when exposed to E2 (Figure 2D).

#### 3.1.4. NRF1 Supports Self-Renewal of Breast Cancer Stem Cells

BCSCs are faced with apoptosis and senescence as a barrier to their self-renewal. The ability of BCSCs to evade apoptosis and senescence contributes to cell reprogramming and stem cell survival, and ultimately progression to a malignant phenotype. Based on this rationale, we investigated the contribution of NRF1 to proliferation, apoptosis, and senescence of a BCSC-like subset (Figure 2E–I).

To examine whether NRF1 expression confers a growth advantage to BCSCs, we measured the proliferative capacity of the BCSC-like subset by assessing cell viability/metabolic activity through reduction of a tetrazolium dye (MTT), cell mass/cell number by binding of the Sulforhodamine B (SRB) dye to basic amino acids of cellular proteins, and BrDU incorporation into newly synthesized DNA. All three methods showed significantly high proliferative capacity based on cell viability/metabolic activity/cell mass/BrDU incorporation in NRF1 overexpressing BCSC-like subsets compared to CD24−/CD44− MCF10A cells (Figure 2E–G). We further determined whether inhibition of NRF1 could reverse the growth advantage seen in a BCSC-like subset. As expected, the suppression of NRF1 inhibited the proliferative capacity of the BCSC-like subset.

To determine if senescence was inhibited by E2 and/or NRF1, senescence-associated β-galactosidase (SA-β-gal) activity was histochemically detected in BCSC-like subsets and vector control cells. Control vector cells were positively stained with SA-β-gal, and E2 treatment significantly reduced its staining. NRF1 overexpressing BCSC-like subsets did not show any SA-β-gal staining in the presence or absence of E2 treatment. These findings indicate that NRF1 suppresses senescence in the BCSC-like subset, which happens to be a major barrier to cell reprogramming and a characteristic of stem cells (Figure 2I).

For apoptosis analysis, BCSC-like subsets were subjected to FACS analysis after FITC Annexin V (*x*-axis) and propidium iodide (PI: *y*-axis) staining. Dead cells stained positive for both FITC Annexin V and PI and these cells had the lowest number of NRF1+ cells (0.42%) compared to vector control (4%) (Figure 2H). The suppression of NRF1 increased the percentage of dead cells staining positive for both FITC Annexin V and PI by 51%. The increase in dead BCSC-like subsets by NRF1 suppression was similar to the effect by a known apoptosis inducer hydrogen peroxide that resulted in death to 71% of BCSC-like subsets. In summary, overexpression of NRF1 helps the BCSC-like subset to evade apoptosis and senescence, which contributes to cell reprogramming and stem cell survival.

#### 3.1.5. NRF1 and/or E2 Treatment Contributed to the Stochastic Re-Programming of Normal MCF10A Cells into Multiple Lineages of Human Breast Cancer Stem/Progenitor Cells

Based on the current knowledge of BCSCs, we know that the CD24−/(low)/CD44+ phenotype alone is not sufficient to characterize the formation of BCSCs. These antigen markers also do not correlate with tumor initiation, invasion, or metastasis. Therefore, we investigated other potential markers for identifying BCSCs along with CD24−/(low)/CD44+. Further characterization of the CD24−/CD44+ BCSC-like subset with other stem cell markers revealed that estrogen-induced breast tumor initiating/cancer stem cells acquire additional phenotypes, such as 49f, EpCam, ALDH1, and CD133 [19]. Results of the FACS studies for BCSC markers are shown in Figure 1D,E and Table 1. Several stem/progenitor cell subpopulations were observed upon E2 treatment. Treatment with a carcinogenic regimen of E2 induced formation of both CD44+/CD49f+ and CD44+/CD49f− cell subpopulations [CD24−CD44+CD49f+ (26%), CD24−CD44+CD49f− (7.5%)]. We only detected CD24−/(low)/CD44− cells, but not CD44+CD49F+ cells in MCF-10A WT or vector treated with vehicle (DMSO). We further sorted the CD24−/(low)/CD44+ cell population for expression of other BCSC markers—ALDH1A1, CXCR4, NRF1, and CD133. E2 generated CD24−CD44+ cells contained different subpopulations: CD24−CD44+ cells with 49f+/−, CD24−CD44+49f+/− with EpCAM+/−, CD24−CD44+49f+/− with ALDH1+/−, and CD24−CD44+49f+/− with CD133.

The CD24−CD44+ subpopulation upon carcinogenic E2 treatment generated as many as six different downstream breast cancer progenitor cell-like subpopulations: CD24−CD44+CD49f+ALDH1+ (9%), CD24−CD44+CD49f+ALDH1- (24%), CD24−CD44+CD49f+ALDH+CXCR4+ (1.3%), CD24−CD44+CD49f+ALDH-CXCR4+ (8%), and CD24−CD44+CD49f+ALDH+CXCR4+NRF1+ (1.3%) (Table 1). The CD24+/CD44+ subpopulation upon carcinogenic E2 treatment produced as many as seven different downstream breast cancer progenitor cell subpopulations (Table 1).

Flow sorting of the stable NRF1 overexpressing CD24−/(low)/CD44+ cell population for the expression of CD49f marker showed induction of two subpopulations—CD24−CD44+CD49f+ (29.78%) and CD24−CD44+CD49f− (34%). Knockdown of NRF1 completely suppressed the induction of both CD49f+/CD49f− cells. Assessment of the expression of other BCSC markers -ALDH1A1, EpCAM, CXCR4, and CD133 in stable NRF1 overexpressing CD44+CD24− and CD44+CD24+ subpopulations showed as many as 10 different downstream breast cancer progenitor cell subpopulations (Table 1).

The combined NRF1 overexpression and treatment with E2 also led to reprogramming of CD24+ and CD24−CD44− subpopulations with multiple breast cancer stem cell signatures containing ALDH1A1, EpCAM, CXCR4, or CD133. The dominant negative form of NRF1 diminished the effects of E2 and/or NRF1 induced acquisition of BCSCs markers (Figure 1D,E and Table 1).

In summary, multiple lineages of human breast cancer stem/progenitor cells were identified by profiling with stem cell markers in NRF1- and/or E2-treated normal MCF10A cells. If the reprogramming of CD24+ and CD24−CD44− cells upon NRF1 overexpression in the presence or absence of E2 treatment occurred in a deterministic manner, the percentage of the BCSC-like subset population would appear to be fixed and be synchronized. On the contrary, we detected multiple BCSC-like subsets supporting the idea of different BCSC fates mediated by stochastic reprogramming upon NRF1 and/or E2 treatment of normal MCF10A cells.

### 3.2. NRF1 and/or E2 Reprogramming Contributed to the Overexpression of Pluripotency Markers OCT4, NANOG, and SOX2

**Figure 3:**
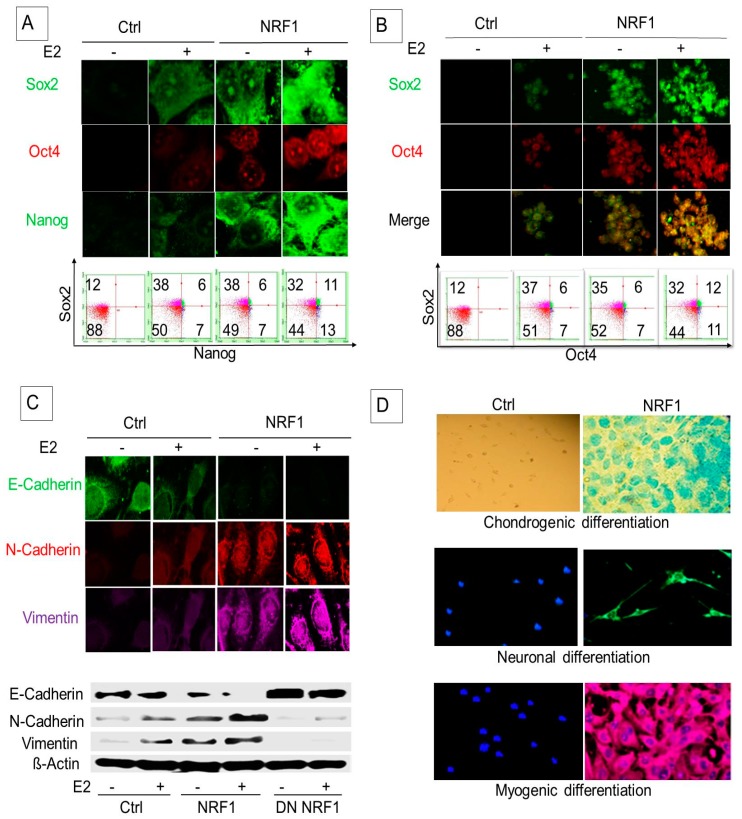
NRF1 and/or E2 re-programming contributed to the overexpression of pluripotency and epithelial-mesenchymal transition (EMT) markers and differentiation of tumor initiating breast cancer cells to other-types of cells. Experimental conditions were the same as Figure 1. (**A**) Immunofluorescent and flow cytometry detections of MCF10A (T) tumor initiating pluripotent stem cells markers (SOX2, Oct4, and Nanog) in transformed breast epithelial cells of MCF10A [MCF-10A (T)] overexpressing vector (control) or NRF1 treated with DMSO and E2. (**B**) Immunofluorescent and flow cytometry detections of pluripotent stem cells markers (SOX2 and Oct4) in transformed breast epithelial cells of MCF10A [MCF-10A (T)] overexpressing vector (control) or NRF1 treated with DMSO and E2. (**C**) Immunofluorescent and Western detections of the expression of EMT markers E-cadherin, N-cadherin, and vimentin in transformed breast epithelial cells of MCF10A [MCF-10A (T)] overexpressing vector (control) or NRF1 treated with DMSO and E2. (**D**) Differentiation of NRF1 tumor initiating stem cells to chondrocytes, neurons, and smooth muscle cells compared to the vector. The vector did not show any differentiation to other cells.

Embryonic pluripotency transcription factors such as OCT4 and SOX2 are important for reprogramming normal breast epithelial cells into BCSCs. Nishi et al. showed that MCF10A cells transfected with OCT4 and SOX2 formed induced pluripotent stem-like cells having a CSC phenotype with high tumorigenicity [21]. BCSC-like subsets derived from MCF10A cells through ectopic NRF1 expression and/or E2 treatment showed increased protein levels of SOX2, OCT4, and NANOG compared to control vector cells (Figure 3A). Our study also showed that NRF1 and/or E2 increased the proportion of SOX2 and OCT4 overexpressing tumor spheroids (Figure 3B).

### 3.3. NRF1 Reprogramming Contributes to a Mesenchymal Phenotype Possessing Multi-Lineage Differentiation Potentials

EMT is considered to be the result of cellular programming of epithelial cells. Complex molecular and biological changes that occur from this programming allow for a mesenchymal phenotype with enhanced migratory capacity, invasiveness, metastatic potential, and drug resistance [22,23]. We postulated that NRF1 re-programming contributes to the acquisition of breast tumor initiating stem (BTIS) cells (breast cancer stem cells) via EMT. We examined the biomarkers of epithelial and mesenchymal phenotypes in BTIS cells by Western blot and confocal microscopy. Overexpression of NRF1 promoted the transition of E2-treated breast epithelial MCF-10A cells to a mesenchymal-like phenotype. We observed the downregulation of epithelial marker E-cadherin and the upregulation of mesenchymal markers, vimentin, and N-cadherin, in NRF1 overexpressing BTIS cells (Figure 3C). In summary, NRF1 seems to be critical for an EMT phenotype. NRF1 acts via loss of E-cadherin and gain of N-cadherin and vimentin in BTIS cells.

One of the properties of mesenchymal BCSCs is that they can differentiate into various connective tissue cells. Therefore, we assessed multi-lineage differentiation potentials of NRF1 mesenchymal BCSCs. We found that NRF1-induced BTICs or BCSC-like subsets differentiated into chondrocytes, neurons, and smooth muscle cells. Differentiated chondrocytes, smooth muscle cells, and neurons stained positive with alcian blue, α-smooth muscle actin (α-SMA), and β-III-tubulin, respectively (Figure 3D). Control vector cells did not show any differentiation to other cells (Figure 3D). These findings suggest that, similar to mesenchymal BCSCs, NRF1-reprogrammed BCSC-like subsets also exhibit multi-lineage differentiation potentials.

### 3.4. Identification of the Mesenchymal-Like “Migrating and Metastasis Initiating” Breast Cancer Stem Cell Phenotype Clone

**Figure 4:**
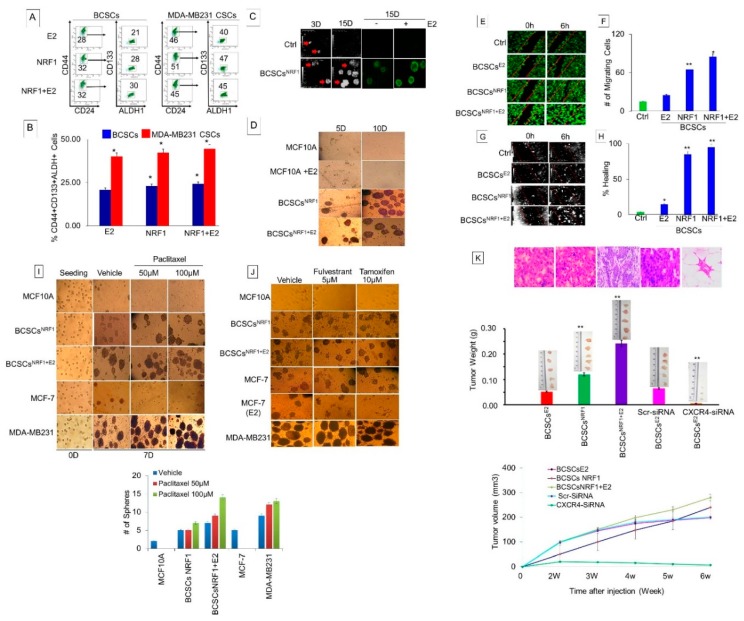
Identification of the mesenchymal-like “migrating and metastasis initiating” breast cancer stem cell phenotype clone. (**A**) NRF1+E2 CD24−CD44+ BCSCs were further flow sorted for the isolation of the CD24−CD44+CD49f+ALDH+CXCR4+CD133+. (**B**) Graph showing NRF1+E2-transformed MCF10A cells displaying CD44+CD49f+ALDH+CXCR4+NRF1+ BCSCs subtype and the most highly aggressive breast cancer cell line—MDA-MB231 (Figure 4A,B). (**C**–**J**) Phenotypic characteristics of CD44+CD49f+ALDH+CXCR4+NRF1+ BCSCs subtype assessed by live imaging of tumorosphere formation, the migration and proliferation potential, and xenograft tumor growth assays; large tumor spheroids were observed by HoloMonitoring (**C**) and confocal microscopy (**D**), as live images of NRF1 and NRF1+E2 BTIC clones compared to the vector control did not form tumor spheroids at 5 and 15 days. (**E**,**F**) This BCSC clone demonstrated a significantly higher potential to close the wound 6 h after initiation of the wound healing assay compared to the vector clone (** *p* < 0.01 and * *p* < 0.05) and higher migration capacity compared to vector clone with and without E2 treatment (**E**–**H**). (**I**,**J**) Effects of anti-estrogens—fulvestrant and tamoxifen; a chemotherapeutic agent—paclitaxel on tumorosphere formation ability of NRF1 overexpressing BTICs or mesenchymal MDA-MB231 enriched with CD44+ +CD133+CXCR4+ clones. (**K**) Xenograft tumor growth NRF1 overexpressing CD44+CD49f+CD133+CXCR4+ BTIC clone after 42 days in (5 out of 5) mice (*p* < 0.001). The upper panel shows macroscopic images of resected tumors, the middle panel shows weight with image of tumors, and the lower panel tumor growth curve of the NRF1 BCSC-like subset and inhibition of tumor growth by silencing CXCR4 expression.

Since NRF1 and/or E2 stochastic reprogramming of CD24+ and CD24−CD44− subpopulations led to the acquisition of multiple distinct BCSC-like subsets, it was necessary to identify and clone the mesenchymal-like “migrating and metastasis initiating” BCSC subset with high potential of forming xenograft tumors to validate the impact of NRF1 in vitro observations by in vivo studies. Patients with triple negative breast cancer (ER−/PR−/HER2−) express CD44+CD49f+CD133/2 in breast tumor tissues, which positively correlate with a stem-cell-derived tumorigenic signature [23]. Tumor-initiating cells enriched for CD133/CXCR4/EpCAM are highly metastatic [24,25]. Molecular signature CD44+ CD133+CXCR4+ has been reported to identify mesenchymal BCSCs found in blood [25]. Similar to MDA-MB231, MCF10A cells are ER−/PR−/HER2− [25]. Flow cell sorting revealed that NRF1 overexpression enriched the cell population with the CD24−CD44+CD49f+ALDH+CXCR4+CD133+ subtype to 30%, while combined NRF1+E2 resulted in 25.20%. These reprogrammed BCSC-like subsets were expressing a molecular signature found in the most highly aggressive breast cancer cell line, MDA-MB231 (Figure 4A,B). The percentage of cells enriched with the CD24−CD44+CD49f+ALDH+CXCR4+NRF1+ BCSC-like subset acquired from NRF1+E2 treatment was approximately 50% when compared to the CD24−CD44+CD49f+ALDH+CXCR4+NRF1+ subtype from MDA-MB231 CSCs (Figure 4B). Therefore, we focused our efforts on the NRF1+CD133+CD44+CXCR4+ BTIC clone and further examined whether NRF1 overexpressing BTICs or the mesenchymal MDA-MB231 clone enriched with the CD44+CD133+CXCR4+ subtype exhibit an invasive phenotype. This was assessed by live imaging of tumorosphere formation, the migration and proliferation potential, and xenograft tumor growth assays. Large tumor spheroids were observed by HoloMonitoring and confocal microscopy, for live images of NRF1 and NRF1+E2 BTIC clones compared to vector control did not form tumor spheroids at 5 and 15 days (Figure 4C,D). NRF1 and NRF1+E2 BTIC clones demonstrated a significantly higher potential to close the wound 6 h after initiation of the wound healing assay compared to vector clone (** *p* < 0.01 and * *p* < 0.05). Additionally, the NRF1 and NRF1+E2 BTIC clones possessed a higher migration capacity compared to the vector clone with and without E2 treatment (Figure 4E–H).

BCSCs are resistant to chemotherapy and hormonal therapy [20,21]. Therefore, we assessed the effects of anti-estrogens—fulvestrant and tamoxifen—and a chemotherapeutic agent—paclitaxel—on the tumorosphere formation ability of NRF1 overexpressing BTICs or mesenchymal MDA-MB231 enriched with CD44+ +CD133+CXCR4+ clones. The NRF1 BCSC-like subset showed resistance to these agents just as observed for breast-cancer-resistant MDA-MB231 cells. As expected, E2-induced MCF-7 cells were sensitive to both anti-estrogens. The tumor sphere forming ability of MCF-7 cells was also completely inhibited by treatment with paclitaxel (Figure 4I,J).

To test the impact of NRF1 signaling in the pathogenesis of estrogen-induced breast cancer, it is critical that we validate our in vitro observations by in vivo studies. To evaluate whether or not NRF1 overexpressing CD44+CD49f+CD133+CXCR4+ BTIC clone was able to form tumors in vivo, we injected these cells into the mammary tissue of SCID mice. The xenograft tumor growth was observed after 42 days in (5 out of 5) mice (Figure 4J; *p* < 0.001). In vector control MCF10A cells, we did not detect any CD24−CD44+ cells by FACS analysis, which is our initial marker of cancer cell stemness (Figure 4J). In addition to using MCF10A vector CD24−CD44− control cells, we also used NRF1 overexpressing cells treated with E2 sorted for NRF1+ with CD24−CD44−CD49f−CD133−CXCR4−ALDH− as another control. These cells did not produce tumors in vivo.

### 3.5. Is NRF1 Protein Enriched in Human Breast Tumor Specimens?

**Figure 5:**
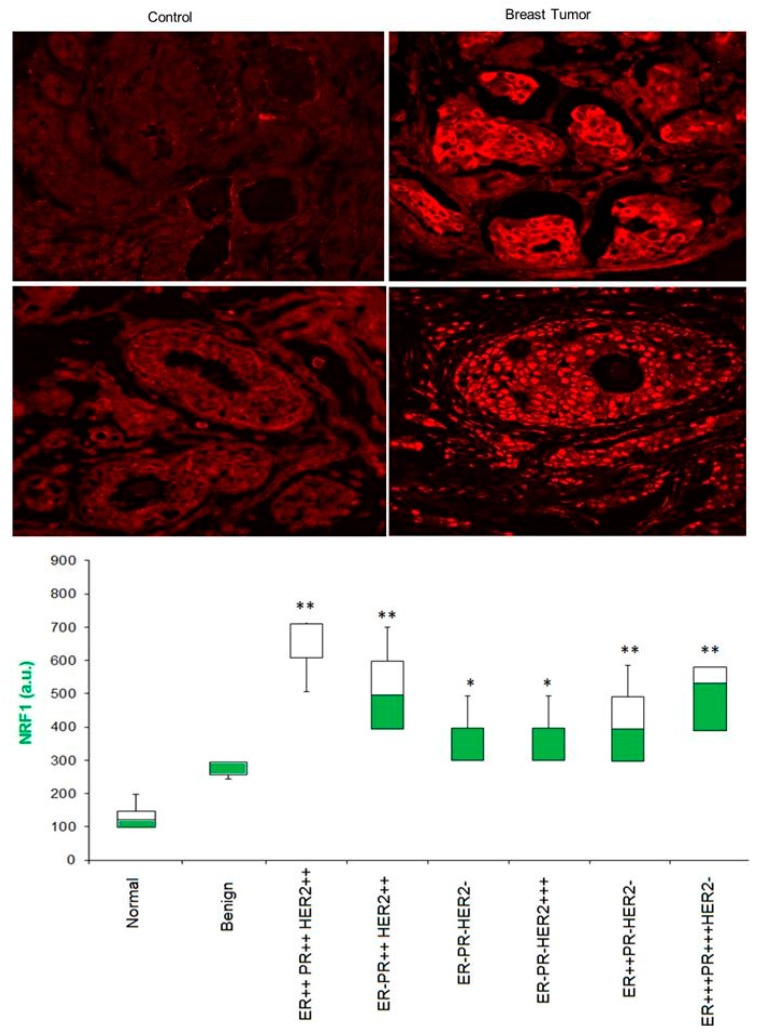
The representative confocal immunofluorescence microscopy image of NRF1 protein expression in breast cancer and box plot showing relative quantitative value of NRF1 intensity in different stages of breast cancer (6 normal, 6 benign, and 138 malignant samples probed with NRF1 antibodies conjugated with Texas Red secondary antibodies ** *p* < 0.01 and * *p* < 0.05).

To determine whether NRF1 protein levels are elevated in breast tumors, we detected NRF1 protein expression using confocal microscopy and dual immunofluorescence staining of the US Biomax breast tumor tissue array with NRF1-specific antibodies paired together with Texas Red-conjugated secondary antibodies. As shown in Figure 5, we detected increas(ed levels of NRF1 protein in breast tumor tissues (both benign and malignant) compared to control tissues (normal breast tissues) (Figure 5). A significantly higher proportion of invasive ductal breast carcinomas overexpressed NRF1 in the nuclei compared to normal breast tissues (27 out of 40 tumors). Most of the NRF1 protein in 10 normal breast tissues appeared in the cytoplasm.

Motifs bound by ELK1, E2F, NRF1, and NFY positively correlate with malignant progression of breast cancer [11]. Similarly, our previous meta-study showed that NRF1 gene expression significantly increases with the progression of breast tumor grades [7]. Therefore, we also examined NRF1 protein expression in tissue samples from the breast cancer subtypes stratified based on the status of ER, PR, and Her2 receptor status. Our finding showed that NRF1 levels were significantly higher in all four subtypes of breast cancers (ER+PR+HER+, ER+PR+HER−, ER−PR−HER+, and ER−PR−HER−) compared with control tissue (*p* < 0.001). ER+PR+HER+ breast cancer specimens had the highest levels of NRF1, followed by other subtypes of breast cancer (Figure 5, box plot in the bottom panel). Our finding is consistent with a recent report showing increased levels of NRF1 protein in breast cancer patients who underwent surgery at the Changhai Hospital of Shanghai, China [26]. Taken together, these findings provide evidence in support of elevated NRF1 protein levels in human breast tumors.

### 3.6. NRF1 Drives Breast Tumorigenesis through Regulating CXCR4 Signaling

**Figure 6:**
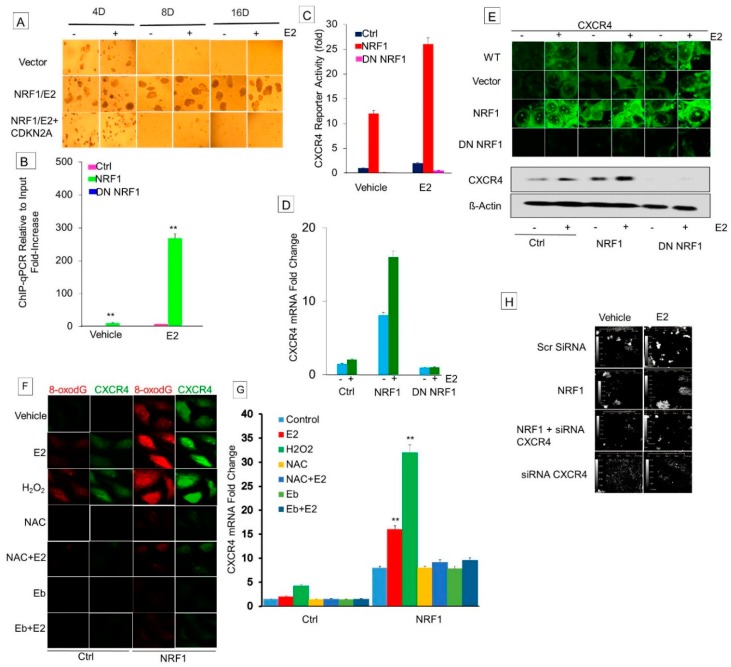
NRF1 drives breast tumorigenesis through regulating CXCR4 signaling. (**A**) Restoration of the expression of p16 gene in BCSCSs significantly reduced tumor sphere formation. NRF1 regulation of CXCR4 in vector, NRF1, and DN NRF1 treated with DMSO (control) and E2 shown by the binding of NRF1 to the promoter of the CXCR4 gene detected by ChIP-qPCR (**B**), reporter assay showing modulation of CXCR4 promoter activity by NRF1 (**C**), mRNA levels of CXCR4 measured by qRT-PCR (**D**), CXCR4 protein detected by immunofluorescence and Western (**E**), ROS dependence detected by immunofluorescence of 8oxodG (**F**), modulation of mRNA levels of CXCR4 by ROS scavengers (**G**), and the representative HoloMonitor microscopy live cells images, showing transfection of SiRNA CXCR4 inhibiting NRF1-induced tumor spheroids (**H**) (** *p* < 0.01 vs. control).

Since the senescent and tumor suppressor p16INK4a gene locus is deleted in the MCF-10A human breast epithelial cells [27], we first determined the effects of restored expression of p16INK4a in the proliferation and self-renewal of BCSCs. Ectopic expression of p16INK4a in BCSCs significantly reduced tumor sphere formation (Figure 6A). This indicates that the restoration of p16INK4a can suppress the self-renewal properties of NRF1 BCSCs. However, this cannot explain how NRF1 drives breast tumorigenesis because p16-deficient MCF-10A cells do not form malignant tumors [11].

We then focused our efforts on the regulation of the metastasis-related CXCR4 gene because it is associated with stem cell acquisition and aggressive growth of breast tumors [14,15,28]. Furthermore, CXCR4 has been reported to be upregulated in breast tumors [29].

#### 3.6.1. Effect of Estrogen and NRF1 on CXCR4 Transcription

CXCR4 is a target of NRF1 that is upregulated in E2-treated MCF-7 cells [30]. Therefore, we determined if NRF1 regulates transcription of CXCR4 in BTICs. We first assessed the binding of NRF1 to the promoter of the CXCR4 gene by ChIP-qPCR. We discovered that NRF1 was bound to the promoter of CXCR4, confirming that its promoter contains the NRF1 DNA element(s) (Figure 6A). NRF1 binding to the CXCR4 promoter was inhibited by DN NRF1. MCF10A cells treated with E2 showed an increase in the mRNA levels of CXCR4 compared to vehicle control cells (Figure 6B). Our findings were consistent with a previous report that showed at the molecular level, E2 and NRF1 regulate CXCR4 gene expression in MCF-7 cells [30,31]. NRF1 overexpression also increased CXCR4 mRNA levels, which was reversed by DN NRF1. We also observed the upregulation of CXCR4 proteins in NRF1+ BTIS, and this effect was reversed by DN NRF1 (Figure 6G). These findings provide a mechanistic explanation for the activation of CXCR4 and corroborated our flow sorting data, showing increased CXCR4 protein levels in NRF1 overexpressing BTIC cells (Figure 1D).

#### 3.6.2. Determine Whether DNA Oxidation Is Necessary for NRF1-Mediated Transcriptional Activation of the *CXCR4* Gene

For the assembly of the transcription initiation complex of the NRF1 target gene, DNA relaxation is required. Oxidized DNA nucleobases such as 8-oxo-7,8-dihydroguanine (8-oxoGua) recruit DNA repair enzymes [e.g., human 8-oxoGua DNA glycosylase 1 (hOGG1)] and topoisomerases, resulting in DNA cleavage and relaxation to help reveal transcription factor binding sites. We observed a significant increase in the mRNA expression of CXCR4 with E2, NRF1 overexpression, or hydrogen peroxide (H_2_O_2_) treatment (Figure 6E). The rise in CXCR4 mRNA levels in E2-, NRF1-, and H_2_O_2_-treated cells seems to be related to the ROS generation because co-treatment with the ROS scavenger ebselen inhibited their effect on CXCR4 expression (Figure 6F). Co-treatment with antioxidants N-acetyl cysteine and ebselen inhibited NRF1-, E2-, or H_2_O_2_-induced expression of CXCR4 when compared to the vector control alone. In addition to the binding of NRF1 to the CXCR4 promoters, NRF1-generated ROS signaling also controls the expression of the CXCR4 gene. These experiments also demonstrate that DNA oxidation is necessary for transcription of NRF1-regulated genes.

#### 3.6.3. Inhibition of Xenograft Tumor Growth of NRF1 BTICs by Silencing the Expression of CXCR4

Next we tested the role of CXCR4 in NRF1-induced breast tumorigenesis. Treatment with CXCR4 shRNA prevented NRF1 plus E2-induced tumorigenicity of CD24−CD44+CD49f+CD133+CXCR4+ALDH+ high NRF1 BTICs (NRF1+E2 scr CXCR4-black compared to NRF1+E2+SiRNA CXCR4-green, *p* < 0.001, Figure 6H). Control mice xenografted with CD24−CD44−CD133−ALDH1− high NRF1+E2 or vector containing CD44−CD24− cells did not produce tumors over time (Figure 4).

These findings together suggest that CXCR4 may play an important role in NRF1- and/or E2-induced breast tumorigenesis.

## 4. Discussion

Whilst the molecular mechanism by which NRF1 may contribute to an individual’s susceptibility to invasive breast cancer is not clear, we are beginning to acquire tantalizing evidence. We for the first time show that NRF1 alone and/or under carcinogenic estrogen-induced stress contributes to generating highly invasive mesenchymal BCSCs via EMT. Our results also showed that heterogeneous BCSC-like subsets are produced by exposure to E2 and/or ectopic expression of molecular risk factor—nuclear respiratory factor 1 (NRF1). The dominant negative form of NRF1 diminished the effects of E2 and/or NRF1 induced acquiring of BCSC-like subsets. If transcriptional programming occurred in a deterministic manner, BCSC-like subsets should appear at a fixed, predictable time, as programming events in all transduced cells would be synchronized. On the contrary, we have identified a mesenchymal CD24−CD44+ BCSC-like subset and as many as 12 downstream breast cancer progenitor cells upon carcinogenic E2 treatment of normal MCF10A cells. Our findings appear to support stochastic acquisition of different CSC fates presumably through NRF1 mediated stochastic transcriptional programming. The same seems to be true in generating heterogeneous ER-BCSCs during the evolution of human breast tumors [32,33]. These findings suggest that estrogenic chemical (17β-estradiol)-dependent stochastic NRF1 transcriptional programming of genetically homogeneous CSC populations results in heterogeneous BCSC-like subsets, with each lineage containing unique surface markers and specific NRF1 molecular signatures. Our results also demonstrate that NRF1 is required for BCSC-like subset self-renewal, and provide evidence supporting the causal role of NRF1 gain in breast tumorigenesis. The above studies provide strong support for our concept that NRF1 plays an important role in the development of breast cancer.

The mechanism by which NRF1 may reprogram adult breast epithelial cells to acquire cancer stem cell properties is not clear. We have recently shown that NRF1 motifs were present in the genes of signaling pathways of all 10 hall marks linked to malignant transformation and progression [13,34,35]. In addition to controlling mitochondrial biogenesis, by activating or repressing the cancer hall mark genes, NRF1 may play a critical role in the acquisition of human cancer hallmark features. A similarity exists in the process of generating tumor initiating cells and induced pluripotent stem (iPS) cells from adult cells. For example, the generation of iPS cells requires the overexpression of embryonic transcription factors OCT4, SOX2, and NANOG, which are considered master regulators of pluripotency. Similarly, BCSCs also require the induction of these pluripotency markers because they are multipotent and shown to differentiate into all cell types of the mammary gland [13,35]. Our results showed that the expression of NANOG, OCT4, and SOX2 were increased by NRF1. The re-activation of embryonic transcription factors is necessary for the acquisition of BTICS or BCSC-like subsets. Recent studies suggest that EMT contributes to the acquisition of cancer stem cell properties [13,14,21]. From ChIP-chip, ChIP-PET, and ChIP-seq analysis, we know not only which genes display altered expression when NRF1 activity is modulated but also which genomic loci are physically occupied by NRF1 [4,5,13,34,35]. NRF1 localizes to several thousand sites in the human genome and may occupy up to 15% of the promoter regions. The percentage of NRF1 promoter occupation seems to be dependent on cell type. Embryonic stem cells have been shown to have roughly 33% of all active genes bound by NRF1 within 1 kb of the transcriptional start site [13,34,36]. The mechanism by which NRF1 drives the expression of embryonic pluripotency transcription factors, OCT4, SOX2, and NANOG necessary for maintaining breast cancer cell stemness remains to be determined. Recently, it has been found that the SOX2 gene lies in an intron of a long multi-exon non-coding RNA SOX2 overlapping transcript (SOX2-OT). SOX2-OT has been reported to positively regulate the expression of SOX2 and OCT4; these two TFs also regulate NANOG expression [37,38]. Our ChIP-Seq experiments revealed that NRF1 is bound to the promoter of the SOX2-OT lncRNA gene (unpublished). SOX2-OT plays a key role in the induction and/or maintenance of SOX2 expression in breast cancer. Since SOX2 participates in the reprogramming of somatic cells to a pluripotent stem cell state and is implicated in tumorigenesis in breast [38], it is possible that NRF1 through SOX2-OT regulates the expression of SOX2 and OCT4 in NRF1+ BTICs.

Our results also showed a mesenchymal-like phenotype from the ectopic overexpression of NRF1 in E2-treated MCF10A cells. It appears that NRF1 transcriptional programming may contribute to the acquisition of BCSC-like subsets via epithelial to mesenchymal transition (EMT) [39,40]. This process allows breast epithelial cells to transform into mesenchymal cells so that they are no longer held in place at the laminin. It involves cadherin downregulation so that cells can detach from laminin and migrate. This was corroborated by the downregulation of epithelial marker E-cadherin and the upregulation of the mesenchymal markers vimentin, N-cadherin, and jerky (JRK/JH8). Histone 3 lysine 4 acetylation (H3K4ac), an epigenetic mark, is a strong predictor of deregulated cancer pathways that lead to the progression from initial transformation to aggressive metastatic phenotypes [41]. H3K4ac enrichment is more dynamic at gene promoters closely linked to EMT (41). The enrichment of acetylated histone H3K56 (H3K56ac) at the co-occupied cluster region of transcription factors OCT4/SOX2/NANOG significantly matched with the NRF1 motif CGCATGCGCR [42]. Since the enrichment of H3K4ac together with H3K4me3 is more reflective of the EMT response pathway in the MDA-MB231 cell line [41,42], it is possible that NRF1-dependent EMT gene(s) are regulated by epigenetic marks facilitated by acetylation and methylation of histone H3 lysines 4 and 56 (H3K4 and 56 ac and/or me3).

To understand the mechanistic aspects of the contribution of NRF1 in susceptibility to the breast carcinogenicity, we focused our efforts on NRF1 motif enriched CXCR4 gene, which is implicated in breast cancer. NRF1 is known to mediate the cellular response to oxidative stress by regulating the expression of genes involved in the cell cycle, DNA repair, cell apoptosis, and mitochondrial biogenesis [13,34]. The crosstalk between mitochondria and nucleus mediated by transcription factors, such as NRF1, plays a significant role in the survival of cancer cells. We do not rule out the contribution of NRF1-mediated transcriptional programming of mitobiogenesis in estrogen-induced cell cycle progression during the development of breast cancer. NRF1 may play a role in estrogen-induced breast cancer development and progression through its interplay with the transcription factors E2F4 and MYC through coupling mitobiogenesis with nuclear replication over the course of the cell cycle progression, indicating an integral link between cell division and mitochondrial biogenesis [13]. Some of the same CXCR4 signaling pathways that are sensitive to ROS levels and E2 are also directly regulated by NRF1 [9]. Recently, CXCR4 has been shown to drive the metastatic phenotype in breast cancer through the activation of MEK and PI3K pathways [14]. In this study, we observed a CXCR4 signaling pathway that not only activated by NRF1 but also was responsive to exposures to both E2 and ROS. The activation of NRF1 was studied in the context of the upregulation of the CXCR4 gene involved in the development of E2-dependent breast cancer. This observation is consistent with our finding of PTEN oxidation, and with earlier reports suggesting that ROS could reversibly modify the redox state of specific cysteine residues in PTPs and make them inactive [8]. These findings have important implications for understanding the molecular mechanisms by which the redox-sensitive molecules AKT or ERK may participate in E2-mediated signaling to NRF1. NRF1 was reported to be a substrate of AKT and activation of AKT controls translocation of NRF1 to the nucleus. This observation is based on a study in which the translocation of NRF1 to the nucleus occurred in PTEN-deficient cells and was abrogated when the PI3K pathway was blocked, inactivating AKT [43]. Our study confirmed that NRF1 is a direct substrate of AKT in the MCF-7 cells. Serine residues 97, 108, and 116 are the major sites in NRF1 that are phosphorylated by AKT. Exposure of MCF-7 cells to E2 not only upregulated NRF1 expression, but it also induced phosphorylation of NRF1 by AKT kinase in a redox-dependent manner. Treatment of cells with ROS modulators or RNAi of NRF1 prevented E2-induced NRF1 expression and phosphorylation. These findings show that AKT catalyzes NRF1 phosphorylation in E2-treated MCF-7 cells through an ROS-mediated signaling pathway. Our current study also showed that silencing of CXCR4 expression reduced BTIC tumor sphere formation and xenograft growth of tumor. To examine the effect of ROS production in the transcriptional activation of NRF1 target gene, CXCR4, we evaluated the effects of ROS scavengers on its expression. Exposure of MCF-7 cells to E2 increased the binding of NRF1 to promoters of these cell cycle genes, and this E2 effect was inhibited by the overexpression of H_2_O_2_ scavenger catalase. To further analyze the mechanism of ROS in regulating cell cycle genes, we studied the effect of ROS scavengers on mRNA expression of NRF1-regulated cell cycle genes and found that E2-induced expression of these cell cycle genes was also inhibited by the overexpression of H_2_O_2_ scavenger catalase. These data suggest that ROS regulate E2-induced transcriptional activation of cell cycle genes through NRF1 modulation. Together, these observations strongly support our concept that the ROS-inducible PI3K-AKT signaling pathway acts as one of the main signal transduction pathways triggering NRF1 activation and subsequent NRF1-mediated transcription of the CXCR4 gene in response to E2 exposure. Our findings suggest that ROS modulators may be useful agents to inhibit the E2-induced tumor phenotype and have the potential for further therapeutic development.

## 5. Conclusions

In summary, the major novel findings of this study illustrate new roles of NRF1 in helping to acquire breast tumor initiating stem-like cells and in regulating EMT and invasiveness of BCSCs, thus opening a new direction of NRF1’s role in breast cancer research. Stochastic heterogeneous BCSCs generation may provide a better understanding of how E2-dependent breast neoplasms depend on the NRF1 network, and this may open new avenues for therapies against breast cancer. The activation of CXCR4 by NRF1 is dependent on ROS formation. Findings of this study provide not only a new understanding for the mechanism of estrogen-induced malignant transformation of MCF10A cells by NRF1, but also important information for the design of a new regime against BCSCs for the prevention and treatment of estrogen-dependent breast cancer.

## Figures and Tables

**Table 1:** Stochastic generation of different cell subpopulations of BCSCs separated by flow sorting by the CD24−CD44+ and CD49f, ALDH1, EPCAM, CD133, CXCR4 and NRF1 phenotypes. 17β-estradiol (E2), NRF1+E2, dominant negative (DN) NRF1 and DN NRF1+E2 treated breast epithelial MCF10A cells were immunostained with CD24 and CD44 antibodies, and subsequently with CD49F, ALDH1, EPCAM, CD133, and CXCR4. The cell subpopulations defined by the CD24−CD44+ and other antigens phenotypes were separated by fluorescence-activated cell sorting (FACS). The percentages (%) shown in the table show the representation of the cell subpopulations in the total transformed by E2 and/or NRF1 or vector (V) transfected parental cell population.

Antigen Markers	V	E2	NRF1	NRF1+E2	DN NRF1	DN NRF1+E2 (%)
CD24+	27.46	*01.46*	00.26	05.36	02.56	02.72
CD24−CD44-	72.54	*71.02*	33.12	27.54	97.46	97.28
CD24+CD44+	ND	*01.46*	02.36	21.54	0.0	0.0
CD24−CD44+	ND	*26.02*	64.12	44.54	0.0	0.0
CD24+CD44+CD49f+	ND	*1.36*	2.36	27.88	0.0	0.0
CD24+CD44+CD49f-	ND	*10.00*	31.00	29.08	0.0	0.0
CD24−CD44+CD49F+	ND	*26.00*	29.78	25.36	0.0	0.0
CD24−CD44+CD49F-	ND	*7.50*	34.08	19.06	0.0	0.0
CD24+CD44+CD49F+EpCAM+	ND	*1.25*	2.36	11.68	0.0	0.0
CD24+CD44+CD49f+ EpCAM-	ND	*4.42*	5.12	11.74	0.0	0.0
CD24−CD44+CD49F+EpCAM+	ND	*5.94*	29.78	25.36	0.0	0.0
CD24−CD44+CD49F+EpCAM-	ND	*20.00*	32	15.36	0.0	0.0
CD24−CD44+CD49f+EpCAM+ALDH1+	ND	*6.0*	30.08	25.46	0.0	0.0
CD24−CD44+CD49f+EpCAM-ALDH1+	ND	*3.0*	2.08	0.0	0.0	0.0
CD24+CD44+CD49f+ALDH+CXCR4+	ND	*1.2*	2.84	12.0	0.0	0.0
CD24+CD44+CD49f+ALDH-CXCR4+	ND	*3.3*	2.24	35.8	0.0	0.0
CD24−CD44+CD49f+ALDH+CXCR4+	ND	*1.3*	30.00	25.20	0.0	0.0
CD24−CD44+CD49f+ALDH-CXCR4+	ND	*8.0*	42.65	5.20	0.0	0.0
CD24−CD44+CD49f+ALDH+CXCR4+CD133+	ND	*1.3*	30.00	25.20	0.0	0.0
CD24+CD44+CD49f+ALDH+CXCR4+NRF1+	ND	*1.2*	2.76	56.60	0.0	0.0
CD24−CD44+CD49f+ALDH+CXCR4+NRF1+	ND	*1.3*	30.59	25.10	0.0	0.0
CD24−CD44+CD49f+ALDH-CXCR4-NRF1+	ND	*0.0*	0.0	15.10	0.0	0.0

Approximately 27.46% of wild-type MCF10A cells were positive for CD24, while a higher percentage of cells (~72.54%) were negative for both CD24 and CD44 markers. MCF10A cells exposed to the carcinogenic E2 treatment regimen showed a shift in the distribution of these molecular markers. Treatment with E2 resulted in 1.46% of the cell population to be CD24+, while 26.02% of these cells were CD24−/CD44+ (Figure 1B,C and Table 1). These two molecular subtypes are typically found in human breast tumor-initiating cells or BCSCs [17]. Undifferentiated basal/mesenchymal cancer stem cells are considered to be CD24−/CD44+, whereas differentiated basal/epithelial cancer stem cells are CD24+/CD44+ [17]. Since our E2 treatment had no effect on the percentage of CD24−/CD44− cells, we infer that the CD24+ MCF10A cells gave rise to these two new phenotypes (CD24+/CD44+ and CD24+/CD44−) during E2-induced cell transformation. ND = Not detected.
